# Can Boron and Cobalt Nanoparticles Be Beneficial Effectors to Prevent Flooding-Induced Damage in Durum and Bread Wheat at Germination and Tillering Stage?

**DOI:** 10.3390/plants14071044

**Published:** 2025-03-27

**Authors:** Antonina A. Novikova, Ekaterina Y. Podlasova, Svyatoslav V. Lebedev, Vyacheslav V. Latushkin, Natalia N. Glushchenko, Kirill A. Sudarikov, Alexander A. Gulevich, Pyotr A. Vernik, Olga V. Shelepova, Ekaterina N. Baranova

**Affiliations:** 1 Federal Scientific Center of Biological Systems and Agrotechnology, The Russian Academy of Sciences, 9 Yanvarya 29, 460000 Orenburg, Russia; katerina.pryakhina@mail.ru (E.Y.P.); lsv@list.ru (S.V.L.); 2 Institute of Development Strategy, 101000 Moscow, Russia; slavalat@yandex.ru (V.V.L.); sudarikov@zolshar.ru (K.A.S.); petr@zolshar.ru (P.A.V.); 3 V. L. Talrose Institute for Energy Problems of Chemical Physics, N.N. Semenov Federal Research Center for Chemical Physics, Russian Academy of Science (INEPCP FRCCP RAS), 38/2, Leninsky Ave, 119334 Moscow, Russia; nnglu@mail.ru; 4 All-Russia Research Institute of Agricultural Biotechnology, Timiryazevskaya 42, 127550 Moscow, Russia; a_gulevich@mail.ru; 5 N. V. Tsitsin Main Botanical Garden of Russian Academy of Sciences, Botanicheskaya 4, 127276 Moscow, Russia; shov_gbsad@mail.ru

**Keywords:** *Triticum durum* Desf., *Triticum aestivum* L., root flooding, pre-treatment, Co, B, wheat flood tolerance

## Abstract

In this study, we investigated the possible effects of cobalt and boron nanoparticles as an inducer of the first stages of development (germination) of hard and soft wheat when simulating flooding as one of the limiting environmental factors. We also investigated the remote effect of treating wheat grains with nanoparticles when flooding was applied already at the tillering stage. To identify the effects of nanoparticles, we used morphometric, biochemical and phenotypic parameters of seedlings and plants of two wheat species differing in origin and the response of these parameters to flooding. Positive effects were found at the germination stage, increasing quantitative indicators under stress. The sensitivity of wheat species to flooding was different, which corresponds to historical and climatic aspects of cultivation. Sensitivity to stress effects associated with loss of germination, decreased growth and photosynthesis was shown for both species. Treatment with cobalt and boron nanoparticles enhanced adaptation to stress and improved photosynthetic parameters, but the encouraging results under stressful conditions were ambiguous and in the case of soft wheat could lead to deterioration of some parameters. Thus, the use of boron and cobalt nanoparticles has potential for reducing productivity under stress, but requires a detailed assessment of the cultivation protocol depending on the genotype.

## 1. Introduction

In recent decades, significant climate change has become increasingly visible worldwide, causing imbalances in the environment [1,2]. As the demand for food increases, there is an urgent need to develop proven, environmentally friendly strategies that can be widely applied to increase crop yields and mitigate climate change. Hard and common wheat (*Triticum durum* Desf. and *Triticum aestivum* L.) are among the world’s major food cereals [3]. Wheat periodically suffers from a complex of abiotic stresses, leading to poorly predictable economic losses. In modern agronomic practice, it is necessary to introduce innovative technologies that can potentially reduce the risk of various abiotic stresses, the most common of which are flooding, drought, and temperature fluctuations [4,5]. Among the new technological advances in nanotechnology, considerable attention is paid to the development and implementation of nanoparticles. These include nanostimulants, nanofertilizers, nanopesticides and nanoherbicides as a promising effector for a controlled and sustainable method of delivering agrochemicals in microdoses to targeted crops. Both positive and negative effects of nanoeffectors on agronomic indicators of plant growth, yield and productivity have been noted; in some cases, this includes changes in the nutritional value of products obtained from food crops [6]. Among abiotic stresses, flooding has the most destructive effect on the growth of sensitive agricultural crops lacking the ability to form aerenchyma or specialize the supply of oxygen to the root system, leading to a limitation of energy processes associated with the respiration of root cells [4,7,8]. Ultimately, this leads either to a significant reduction in yield [9] or to the death of plants, causing the need for compensatory sowing, provoking additional risks and increasing costs [10]. Different growing conditions of hard and soft wheat can cause differences in their response to this type of stress, so the mechanisms and reactions can differ significantly. Sometimes, flooding occurs as a result of flood waters or prolonged rains; less often, there may be river floods or groundwater rises as a result of water inflows associated with climatic cataclysms in a neighboring region [11]. The mechanism of resistance of hard and soft wheat plants to flooding is the subject of study of many researchers and is not completely clear. A number of studies have analyzed possible adaptive strategies used to compensate for damage associated with cell death, especially of the meristem, caused by hypoxic and anoxic stress [12].

Nanotechnology has significant potential to enhance agricultural productivity through non-traditional means. Recently, this technology has gained momentum as a possible solution to reduce the adverse effects associated with various stresses, including water scarcity, salinity, and flooding [13,14,15]. The advantages of nanotechnology lie in the unique properties exhibited by a diverse spectrum of nanoparticles and their influence on plant growth, and especially on the mechanism of resistance to various abiotic stresses. Nanoparticles are very small molecular clusters with diameters ranging from 1 to 100 nm [16]. The most important difference between nanomaterials and other materials is their increased relative surface area and quantum effects. Their extremely small size often results in diverse and unique physicochemical properties such as varied particle morphology, large surface area, flexible pore size and increased reactivity compared to their bulk material counterparts [17,18]. In agriculture, nanoparticles are used to regulate processes such as plant growth and development, increasing crop yields and preventing various stress factors. The most common forms of nanoparticles are carbon nanomaterials, metal and metal oxide nanoparticles, while the influence of macro- (Na, K, P, Ca) and microelements (Fe, Cu, Zn, Mo, as well as their oxides) in the form of nanoparticles is most often studied. It has been shown that they actively influence seed germination, physiological processes, including photosynthesis, activity of antioxidant enzymes, metabolism of proline, proteins and carbohydrates, which contributes to an increase in biomass, and length of shoots and roots of plants [19,20,21]. It was also demonstrated that TiO_2_ nanoparticles increased resistance to various stresses (drought, salinity and water) by influencing various processes such as accumulation of malondialdehyde and superoxide radicals, while simultaneously inducing the activity of antioxidant defense enzymes [22]. Cobalt is a metal that promotes nitrogen fixation and chlorophyll formation, but at the same time has potential mutagenic and carcinogenic properties [17,23]. Boron in young plants is responsible for activating growth; its deficiency leads to slower growth and pollen germination. It has been shown that foliar spraying of lettuce with calcium borate nanoparticles promotes better accumulation of boron in shoots and roots (accumulation efficiency exceeds the control by 2.56 times) [20]. The potential of nanoparticles of these metals as nanopreparations for preventing damage associated with anoxia and hypoxia has not been previously investigated.

The effect of elements such as boron and cobalt, which actively participate in the physiological processes of plants throughout ontogenesis, on the growth and morphometric parameters of plants has been studied to a lesser extent [24,25]. The production of nanoparticles is generally a fairly routine method, but requires control of the size and quality of the preparations to ensure high-quality penetration and adsorption on plant objects.

In this study, we investigated boron and cobalt nanoparticles and their putative protective effects on two wheat cultivars under flooding induction in a closed system. We used seedlings exposed to excess moisture as seedling flooding models, with imbibition, coleorhiza emergence, and germination limited by unfavorable conditions. To study field flooding conditions, we used a pot culture modeling root flooding at the tillering node level of cultivated plants.

## 2. Results

**Figure 1:**
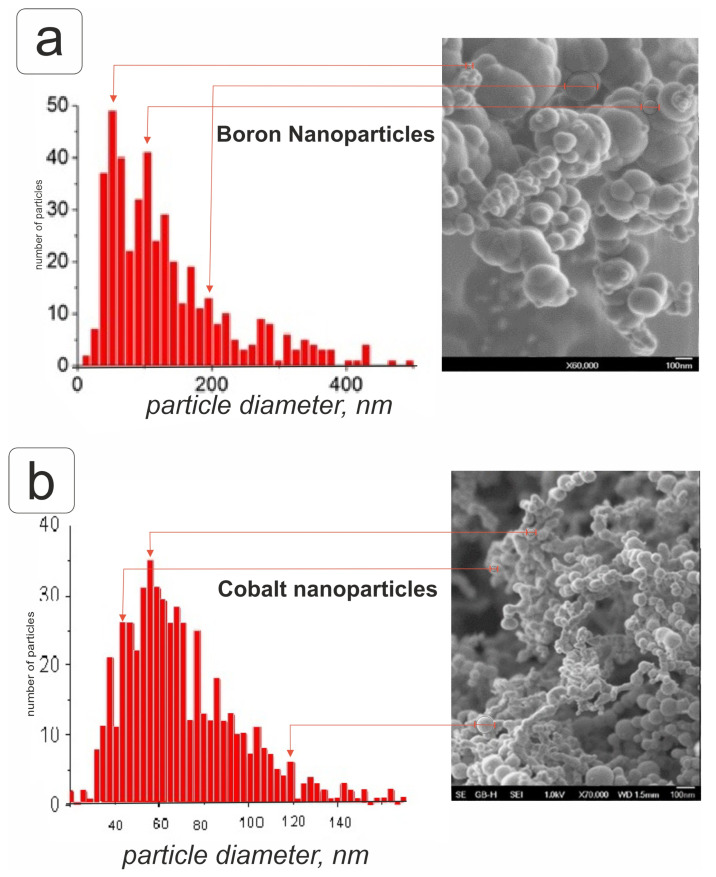
Size distribution curve of boron (**a**) and cobalt (**b**) nanoparticles; scanning electron microscopy images of dried accessions of boron and cobalt nanoparticles.

Nanoparticles were generated by high-temperature condensation using the Migen-3 device [26]. The study of the physicochemical characteristics was carried out in the Laboratory of Nano- and Microstructural Materials Science of the Talrose Institute of Energy Problems of Chemical Physics of the Russian Academy of Sciences (Moscow, Russia). After passivation, when replaced with atmospheric gas, a film of boron and cobalt oxide was formed, respectively, with the oxide phase being at least 5%. The nanoparticles had the following characteristics: the average diameter of cobalt nanoparticles was 63.6 ± 0.6 nm, and the size of the boron particles was slightly larger than 100 nm. Of these, 75% were smaller than 100 nm, 20% were between 100 and 134 nm, and about 5% were larger. Analysis of a 10 mg/mL boron nanoparticle suspension on a Microtrack Nanotrack Wave II (Microtrac Inc., Montgomeryville, PA, USA) showed that the hydrodynamic diameter of the boron nanoparticles was 85.2 nm (100%), and the zeta potential was monodisperse with a single peak at 41.7 mV. Boron nanoparticles had an amorphous structure with a boron content of 92.5% (Figure 1a). In cobalt nanoparticles, the crystalline metallic phase accounted for 95% (Figure 1b).

**Figure 2:**
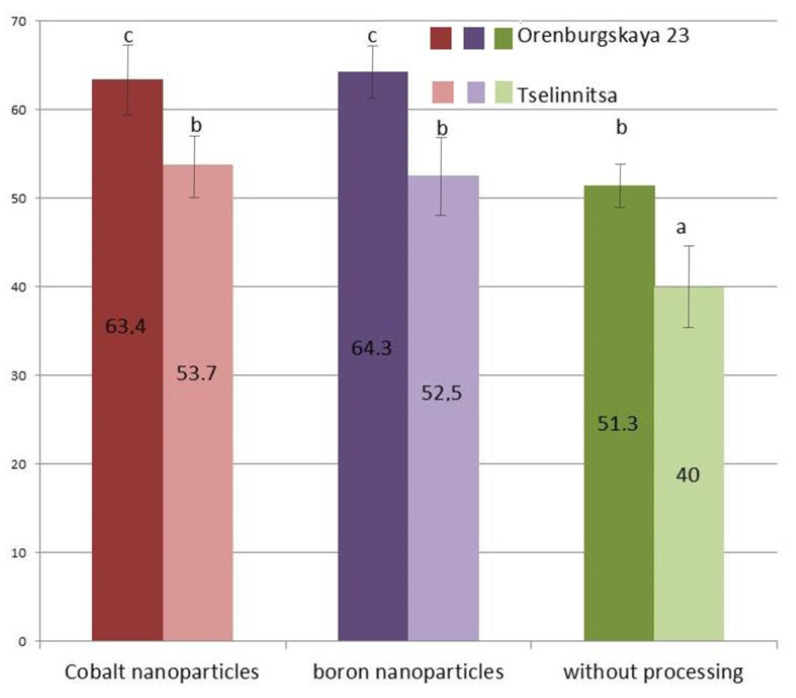
Index of resistance of seeds of spring soft wheat, i.e., the cv Orenburgskaya 23, and spring hard wheat, i.e., the cv Tselinnitsa, to flooding, reflecting the ratio of seed germination with and without flooding. Dark color—cv Orenburgskaya 23, light color—cv Tselinnitsa. The data were obtained in triplicate. Different letters indicate significant differences between treatment, analyzed by Duncan’s tests with the corresponding errors (*p* ≤ 0.05).

The resistance index (an integral indicator of seed resistance to flooding) increases significantly when using boron and cobalt nanoparticles (Figure 2). Thus, for soft wheat, the resistance index after seed treatment with cobalt nanoparticles increased by 13% compared to the control, and by 12.1% when treated with boron nanoparticles. The difference compared to the control (without treatment) remains for both the Orenburgskaya 23 soft wheat and the Tselinnitsa hard wheat. According to the test data, the durum wheat variety was less resistant to flooding than the soft wheat variety (by 9.7–11.8% in different variants of the two-factor experiment (i.e., with different variants of nanoparticle treatment)). The difference in the effectiveness of grain treatments with different types of nanoparticles was insignificant, i.e., resistance to flooding increased under the action of both cobalt and boron nanoparticles.

**Figure 3:**
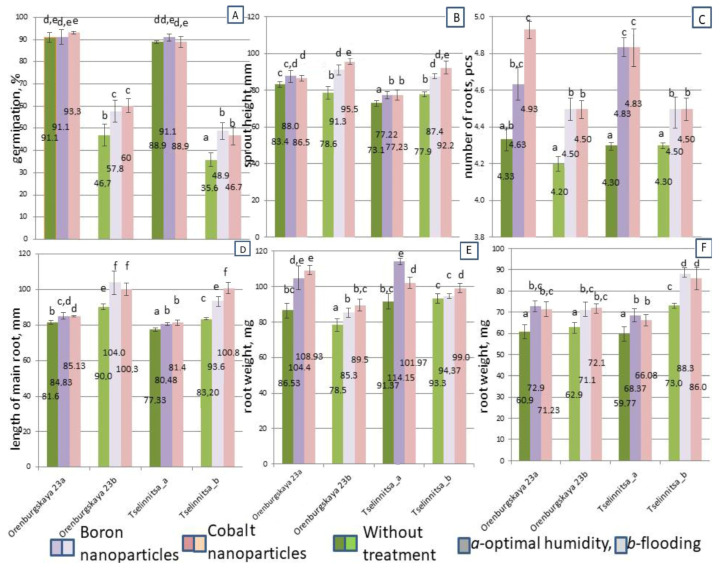
Physiological indicators of seedlings of the cv Orenburgskaya 23 soft wheat and the cv Tselinnitsa hard wheat under flooding. The plants were at the seedling stage (05–09 on the BBCH scale). (**A**)—% germination; (**B**)—seedling height; (**C**)—root numbers; (**D**)—main root length; (**E**)—root weight; (**F**)—seedling weight without roots. Different letters indicate significant differences between treatments, analyzed by a Duncan’s tests with the corresponding errors (*p* ≤ 0.05).

Juvenile stages of germination are a sensitive point for viability under abiotic stresses, particularly flooding. Preventing damage in this way can reduce losses from seedling mortality, affecting final crop productivity. When flooded, an almost twofold drop in germination was observed for soft wheat and a 2.5-fold drop for hard wheat (Figure 3A). The application of nanoparticles contributed to the reduction in the loss of viable seedlings during germination, allowing the viability of more than 57.8% (Co) and 60% (B) of seeds to be preserved in the Orenburgskaya 23 soft wheat and 46.7% (B) and 48.9% (Co) in the Tselinnitsa hard wheat (Figure 3A). The height of seedlings in surviving soft wheat plants decreased, while in hard wheat, on the contrary, it increased when flooded (Figure 3B). Treatment with both boron and cobalt nanoparticles resulted in increased shoot growth, with the effect of boron being greater (Figure 3B). Root growth initiation under these conditions stimulated by nanoparticles was reduced in soft and hard wheat, but was higher than control values (Figure 3C). Both boron and cobalt nanoparticles resulted in enhanced root growth initiation under flooding, with boron having a greater effect (Figure 3C). The length of the main root increased during flooding (Figure 3D). Nanoparticles induced primary root growth both in control and more significantly under flooding (Figure 3D). However, root weight decreased in bread wheat but not in durum, where there was a small increase (Figure 3E). Plants treated with nanoparticles had reduced growth, but it was more intense than that of untreated control plants (Figure 3E). At the same time, the weight of above-ground organs increased slightly in soft wheat and showed a significant increase in hard wheat (Figure 3F). The role of nanoparticles in weight gain under flooding conditions was noted for durum wheat, while for soft wheat, increased gain was observed after treatment with both boron and cobalt nanoparticles. However, a decrease in weight from the flooding effect was observed after cobalt treatment, but not after boron treatment (Figure 3F).

**Figure 4:**
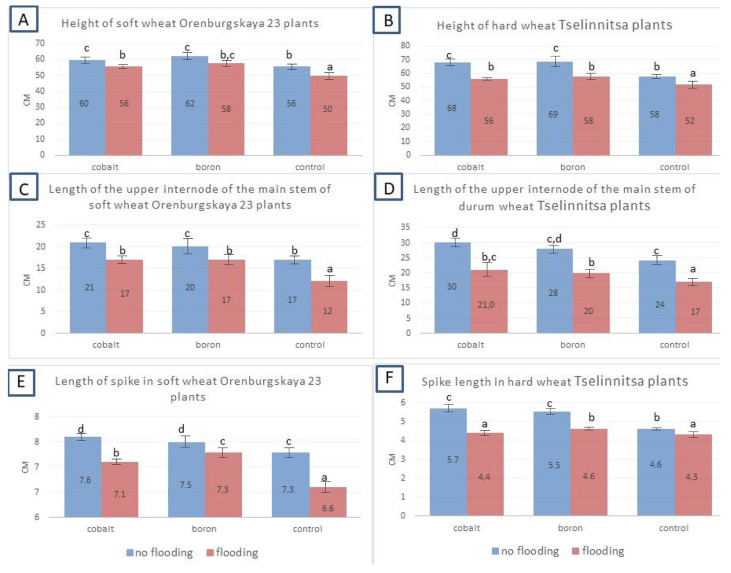
Indicators of plant height and length of the upper internode of the soft wheat cv Orenburgskaya 23 and the hard wheat cv Tselinnitsa under flooding. Different letters indicate significant differences between treatments, analyzed by Duncan’s tests with the corresponding errors (*p* ≤ 0.05).

The data presented in Figure 4 showed that in the full-cycle experiment (growing in a climate chamber—synergotron) under flooding in all variants (with and without treatment with nanoparticles), the plant height (by 6.5–10.7% for soft wheat and 10.3–17.6% for durum) and the length of the upper internode (by 15–29% and 28.5–30%, respectively) decreased. Thus, both traits can be considered diagnostic in assessing resistance to waterlogging, with the upper internode length indicator decreasing more strongly under unfavorable conditions. Grain treatment with nanoparticles contributed to an increase in plant height by 7.1–11.6% for soft wheat and by 16.7–25% for durum, and the length of the upper internode by 11.7–41.7% and 16.7–25%, respectively. No specific pattern has been established in the comparative effectiveness of cobalt and boron nanoparticles in terms of plant height and upper internode length.

**Figure 5:**
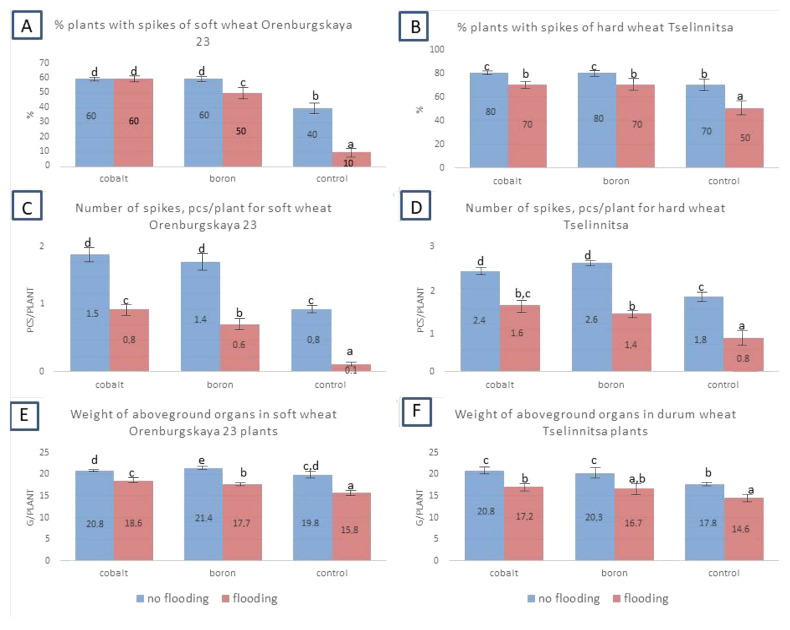
Indicators of the morpho-physiological state of wheat plants after 20 days of flooding: the proportion of plants with spikes (%) and the number of spikes per plant in soft wheat cv Orenburgskaya 23 and hard wheat cv Tselinnitsa during flooding. Different letters indicate significant differences between treatments, analyzed by Duncan’s tests with the corresponding errors (*p* ≤ 0.05).

Under flooding conditions, the number of spikes in wheat plants sharply decreased (Figure 5), for example, in soft wheat, it decreased almost 2 times. The number of spikes increased significantly when grains were treated with nanoparticles in both flooded and non-flooded variants. To a somewhat lesser extent, indicators such as the number of plants with spikes (in percent of all experimental plants) and the length of the spike changed under flooding, but the main patterns remain the same. Thus, treatment with cobalt and boron nanoparticles resulted in a greater number of spikes per plant and an increase in the length of the spike. When flooded, all these indicators decreased.

**Figure 6:**
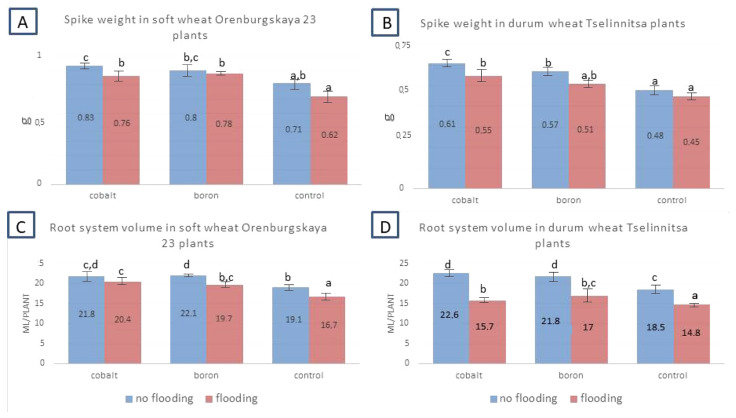
Indicators of above-ground weight, weight of spikes, volume of the root system in plants of soft wheat cv Orenburgskaya 23 and hard wheat cv Tselinnitsa under flooding. Different letters indicate significant differences between treatments, analyzed by Duncan’s tests with the corresponding errors (*p* ≤ 0.05).

The resulting indicators of plant growth are the weight of the plant and its individual organs. The total above-ground weight during flooding decreased by 10.6–20.2% for soft wheat (with different treatment options with nanoparticles) compared to the control and by 17.3–18% for hard wheat (Figure 6). When flooded, this weight decreased to a lesser extent (by 2.5–12.7% and 6.3–10.5% for soft and hard wheat, respectively). The indicator of the root system volume also proved to be informative. In hard wheat plants, when flooded, it decreased by 20–35%, and in soft wheat by 6.2–12%. Thus, when flooded, plant growth was weakened, and plants accumulated less biomass of both above-ground organs and the root system.

Grain treatment with nanoparticles had a positive effect on plant growth and development both with and without flooding. In soft wheat plants, after treatment, the aboveground weight increases by 5.1%, and in hard wheat by 14–17.8%. The weight of the spike increased by 12.7–25.8% and 13.3–27.1% and the volume of the root system by 14.1–22.2% and 6.1–22.2%, respectively.

Comparing the data in Figure 4, Figure 5 and Figure 6, we can conclude that the main morphological and biometric indicators of plants change mainly in the direction of decrease when exposed to a stress factor, flooding. At the same time, treatment of grains with cobalt or boron nanoparticles contributed to increased stress resistance, although it did not completely eliminate physiological disorders and delays in growth and development. Nanoparticles proved to be effective not only in the presence of flooding, but also in its absence, i.e., they increased the weight of the biomass and increased the value of other growth parameters.

**Figure 7:**
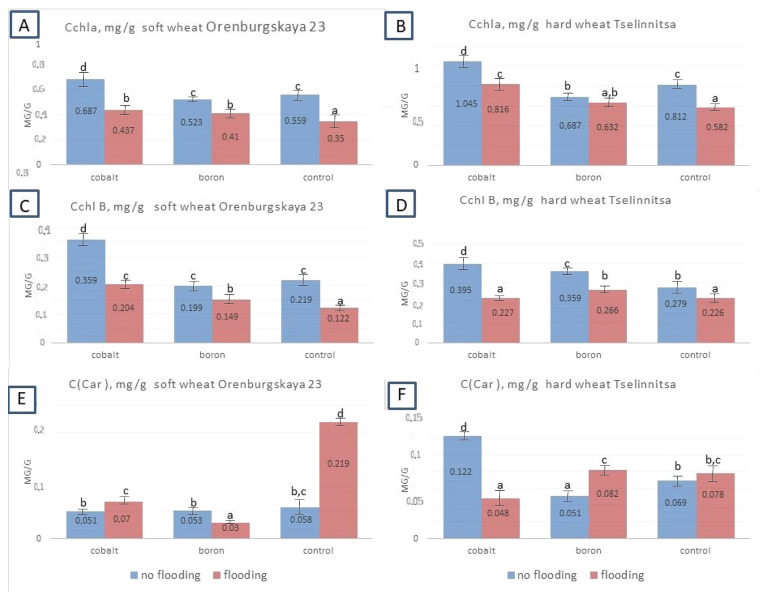
Content of chlorophyll a and b, as well as carotenoids in the leaves of soft wheat cv Orenburgskaya 23 and hard wheat cv Tselinnitsa during flooding. Different letters indicate significant differences between treatments, analyzed by Duncan’s tests with the corresponding errors (*p* ≤ 0.05).

The content of chlorophyll a and chlorophyll b showed the same trend as in the analysis of morphological and biometric parameters of plants: a decrease in content in the variants with flooding compared to the control and an increase in content in the variants with grain treatment with boron and cobalt nanoparticles (Figure 7). Similar data were obtained for both the hard wheat cv Tselinnitsa and the soft wheat cv Orenburgskaya 23. At the same time, in most variants, the opposite tendency for accumulation of carotenoids was noted. Their quantity, on the contrary, increased after exposure to a stress factor (flooding).

**Figure 8:**
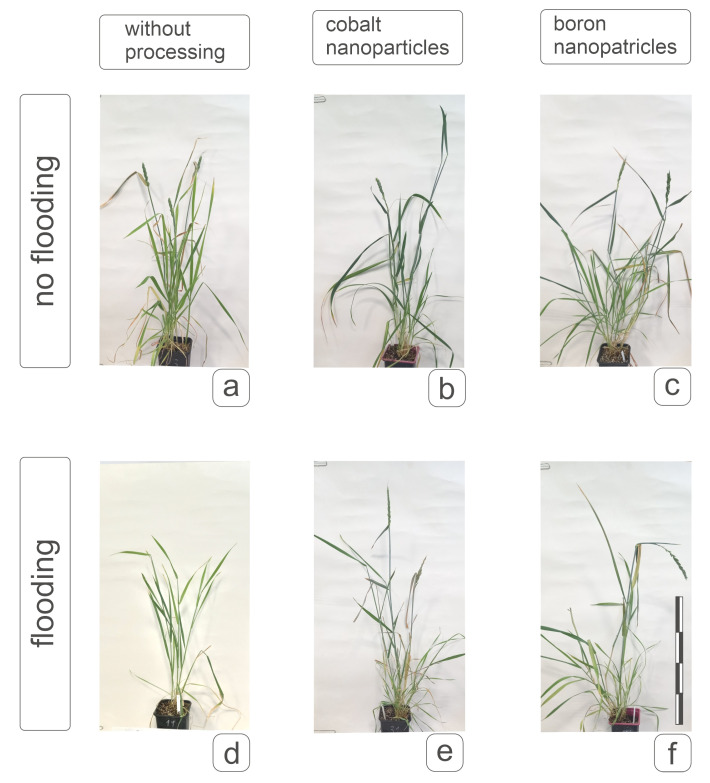
The effect of pre-treatment of grains with boron and cobalt nanoparticles on the manifestation of the phenotype of spring soft wheat cv Orenburgskaya 23 under control conditions and with 20-day root flooding at the tillering stage. Designations: (**a**,**d**)—control plant; (**b**,**e**)—plant from seeds treated with Co nanoparticles; (**c**,**f**)—plant from seeds treated with B nanoparticles. (**a**–**c**)—plants not subjected to flooding; (**d**–**f**)—plants after flooding. Measuring bar, division 10 cm.

Phenotypic characteristics of plants obtained after 20-day flooding at the tillering stage are shown in Figure 8. A clear inhibition of the development of shoots formed at the tillering stage was observed both for plants without nanoparticle pre-treatment of grains and for plants obtained from grains treated with nanoparticles (Figure 8d–f). It is obvious that all plants have reached the earing stage and formed a spike; flag leaves and typical elongated shoots are visible. However, in plants without nanoparticle treatment, flooding caused a slowdown in growth processes and biomass accumulation, and spike emergence was identified in single cases (Figure 8d). On the other hand, the action of boron and cobalt promoted spike emergence on the main shoot and partially on the lateral shoots (Figure 8e,f). It is also worth noting that a positive effect on weight gain was observed under the action of boron nanoparticles without the effect of flooding and a decrease in weight under the action of cobalt nanoparticles, which, however, did not affect the flag leaf responsible for the nutrition of the spike. The action of nanoparticles in the absence of flooding caused some juvenilization of development, but did not prevent the formation of lateral shoots in soft wheat plants of the cv Orenburgskaya 23, showing the absence of obvious advantages of using pre-treatment under control conditions (Figure 8b,c), but maintaining the possibility of more effective generative potential under stress conditions (Figure 8e,f).

**Figure 9:**
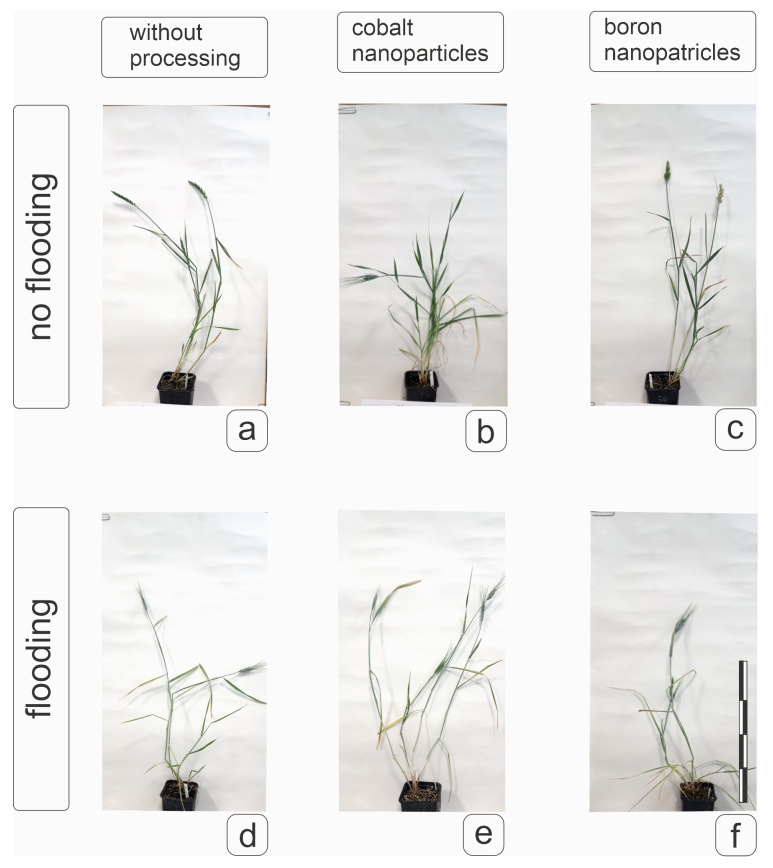
The effect of pre-treatment of grains with boron and cobalt nanoparticles on the manifestation of the phenotype of spring hard wheat of the cv Tselinnitsa under control conditions and with 20-day root flooding at the tillering stage. Designations: (**a**,**d**)—control plant; (**b**,**e**)—plant from seeds treated with Co nanoparticles; (**c**,**f**)—plant from seeds treated with B nanoparticles. (**a**–**c**)—plants not subjected to flooding; (**d**–**f**)—plants after flooding. Measuring bar, division 10 cm.

Phenotypic characteristics of spring hard wheat plants of the cv Tselinnitsa variety did not reveal a positive effect of pre-treatment with cobalt and boron nanoparticles on plant habitus and weight (Figure 9b,c), while pre-treatment with cobalt had a noticeable inhibitory effect on plant growth and weight (Figure 9b), but not on the condition of the leaves. After flooding, plants obtained from grains treated with nanoparticles, although demonstrating a decrease in weight detected under optimal conditions, formed flag leaves worse than the same plants under normal conditions, but better than the control ones. This could significantly affect the compensation for the loss of productivity against the background of such an unfavorable factor as flooding (Figure 9e,f). It can be noted that the plants of the cv Tselinnitsa differed in their reaction from soft wheat plants to flooding and pre-treatment with nanoparticles, since these factors did not cause inhibition of the development of formed shoots (Figure 8 and Figure 9).

**Figure 10:**
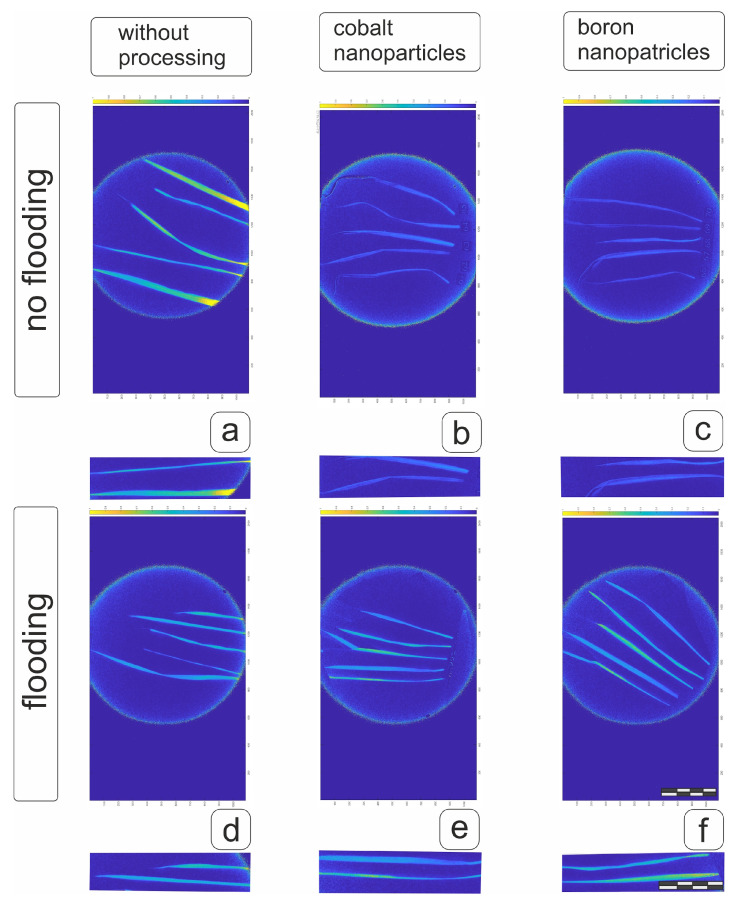
NDVI of separated flag leaves of soft wheat cv Orenburgskaya 23 after pre-treatment of grains with boron and cobalt nanoparticles under normal conditions and with 20-day root flooding at the tillering stage. Designations: (**a**,**d**)—control plant; (**b**,**e**)—plant from seeds treated with Co nanoparticles; (**c**,**f**)—plant from seeds treated with B nanoparticles. (**a**–**c**)—plants not subjected to flooding; (**d**–**f**)—plants after flooding. Measuring bar, division 5 cm.

Visualization of NDVI values on fresh separated flag leaves of the main spike indicated that pre-treatment with boron and cobalt nanoparticles had a prolonged inhibitory effect at the heading stage (Figure 10b,c). However, the decrease in NDVI in flag leaves after 20 days of flooding (Figure 10d) was partially offset by pre-treatment of grains with both boron and cobalt nanoparticles, which would have a positive effect against the adverse effects of flooding (Figure 10e,f and Figure S1).

**Figure 11:**
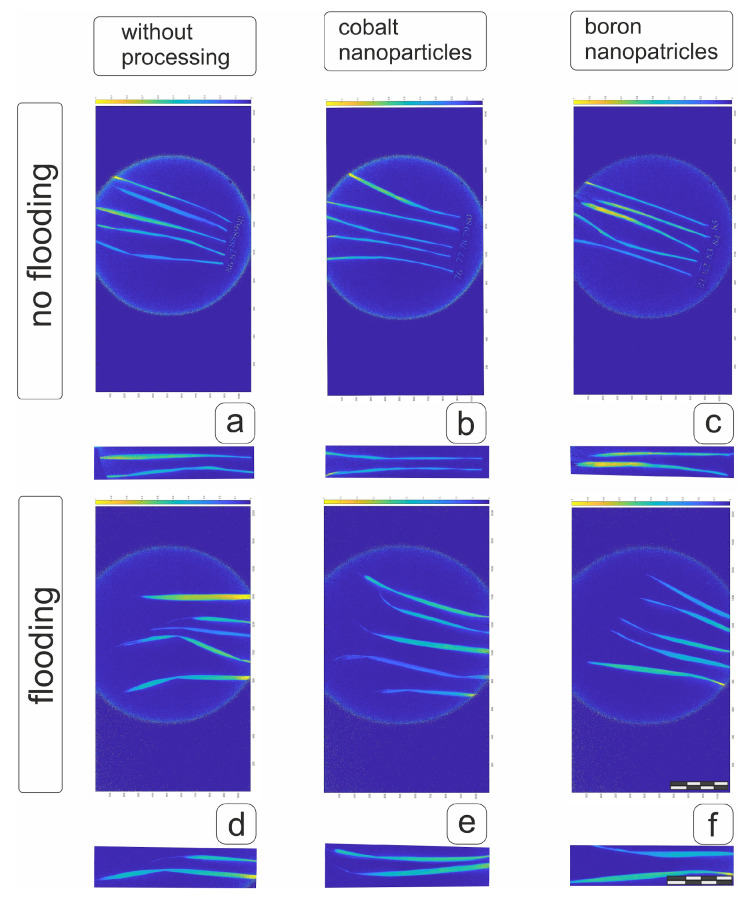
NDVI of separated flag leaves of durum wheat of the cv Tselinnitsa after pre-treatment of grains with boron and cobalt nanoparticles under control conditions and under 20-day root flooding at the tillering stage. Designations: (**a**,**d**)—control plant; (**b**,**e**)—plant from seeds treated with Co nanoparticles; (**c**,**f**)—plant from seeds treated with B nanoparticles. (**a**–**c**)—plants not subjected to flooding; (**d**–**f**)—plants after flooding. Measuring bar, division 5 cm.

Visualization of the NDVI values when assessing flag leaves separated from vegetative plants (Figure 11) demonstrated a positive effect of boron nanoparticles on the condition of the flag leaf of the hard wheat cv Tselinnitsa under control conditions (Figure 11c), while the effect of cobalt nanoparticles worsened the condition. At the same time, some of the flag leaves were only slightly inferior to the control indicators, while some demonstrated significantly worse indicators (Figure 11b and Figure S2). Flooding had a positive effect on the NDVI values of flag leaves in both plants obtained from untreated grains and those pre-treated with cobalt and boron nanoparticles (Figure 11d–f). Given the increase in leaf area, it can be assumed that flooding for cv Tselinnitsa wheat plants will have a positive effect on overall productivity.

## 3. Discussion

Today, the use of nanoparticles is becoming an increasingly popular method for the application of drugs with high efficiency. Some questions arise regarding the prolonged action of such treatments. Nanoparticles can have both direct and indirect effects. The origin of nanoparticles can also be different. Thus, there are examples of effective synthesis using physical effects such as laser, high voltage, pressure, as well as chemical synthesis using physical mechanisms such as the ability to crystallize under certain conditions. Particular attention is also paid to the production of green nanoparticles [27], limited by plant metabolism [28,29]. Recently, opportunities have emerged related to the production of nanoparticles by correcting enzymatic systems through genetic modifications [30,31]. The chosen comparison model between soft and hard wheat is justified by the peculiarities of the origin of these two types of wheat [32]. Previously, we identified differences in the response of these species to stress [33]. Nanoparticles can have both positive and negative effects on plant development. In particular, the toxic effects of cobalt have already been discussed, and its use requires consideration of potential benefits and harms [34,35]. It has been shown that the effect can vary depending on the size, concentration and method of nanoparticle production [36]. Boron is considered to be potentially safer and more useful, but its use also continues to be the subject of study [25,37,38].

Flooding had a strong negative effect on germination rates and morphometric parameters of grains (Figure 4). It was established that the most sensitive parameter of grain resistance to flooding was the germination rate, which decreased during flooding by 33.3–48.7% for soft wheat and by 42.2–53.3% for hard wheat. The germination of hard wheat grains during flooding decreased to a greater extent than that of soft wheat, which indicates the lower resistance of the studied hard wheat variety. Due to the high sowing properties of the grains (91.1% germination for soft wheat and 88.9% for hard wheat), treatment with nanoparticles in the control variant (without flooding) had little effect on germination. Germination increased by only 2.2%. However, after flooding, an effect of increasing stress resistance after treatment with nanoparticles by 11.1–13.3% was noted. The difference in the effectiveness of cobalt and boron nanoparticles was slight, i.e., all of these nanoparticles partially mitigated the negative impact of flooding (but did not completely eliminate the effect of the stressor). Other biometric indicators (number of roots, length of the main root, weight of roots and above-ground part) were less informative in determining sensitivity to flooding, although in most variants these indicators decreased during flooding compared to the control. However, in some variants, for example, in hard wheat, the above-ground weight of shoots and the length of the main root were higher in the flooded variant. This can perhaps be explained by the elimination of weak shoots during the germination period under the influence of flooding, the result of natural selection for resistance.

It should be noted that treatment with nanoparticles had a stimulating effect on the growth and development of wheat seedlings in both flooded and control (without flooding) variants. As shown earlier, seed germination after treatment with nanoparticles increased significantly only in the flooded variants, while in the control variant, the increase was insignificant. Pre-treatment with nanoparticles has a long-lasting effect [35,39] and can have both positive and negative effects on potential productivity. Therefore, it is necessary to clearly assess the risks of application technologies that are effective in the early stages (germination) and their delayed aftereffects at subsequent stages of development (tillering and heading).

In general, the obtained effects can be summarized as follows.

By Factor A. Flooding

For most parameters studied, hard wheat plants were less tolerant to flooding at the germination stage than soft wheat plants. However, at the heading stage, the flag leaf, which provides nutrition to the developing kernels, demonstrated positive dynamics of the NDVI in hard wheat and negative dynamics in soft wheat in plants subjected to 20-day flooding. This may indicate differences in sensitivity to the adverse effects of flooding at these two stages (germination and tillering).

Different species and cultivars may have different susceptibility to root flooding, but general depression is a characteristic feature of flooding of wheat [40]. The most sensitive parameter to flooding at the grain germination stage is germination and the associated resistance index. Other biometric indicators (number of roots, length of the main root, weight of roots and aboveground part) were less informative.

In some variants, the weight of the above-ground shoots and the length of the main root were higher in the flooded variant. It is possible that after exposure to flooding at the germination stage, the most powerful kernels and seedlings overcome the suppression, and a certain kind of adaptation occurs in the surviving seedlings.

Flooding of plants at the tillering stage can greatly hinder development and transition to heading [41,42]. Flooding of roots at the tillering stage for 20 days resulted in a decrease in the activity of growth processes in both soft and hard wheat plants. The main morphological and biometric parameters of plants (plant height, length of the upper internode, volume of the root system, weight and length of the spike, number of spikes, aboveground weight) changed towards a decrease after exposure to a stress factor (flooding).

The content of chlorophyll a and chlorophyll b shows the same trend as when analyzing the morphological and biometric indicators of plants: a decrease in indicators during flooding. However, for carotenoids, the opposite trend was observed in most cases. This probably occurs due to the specificity of adaptation processes, where carotenoids play the role of adaptogens. Flooding has a significant impact on the efficiency of photosynthesis [43,44,45] and the maintenance of high levels of productivity [46].

By Factor B. Nanoparticles

The effect of nanoparticles, like any factor applied to plants, can have both positive and negative consequences [47]. A positive effect on photosynthesis may be noted when respiration is damaged or, for example, when exposed to cytotoxicity [48]. Treatment with boron and cobalt nanoparticles contributed to an increase in the stress resistance of both soft and hard wheat plants for almost all studied indicators at the germination stage. The positive effect of boron on plant development under stress loads has been well studied and supported by many studies [49], but there are also data that require clarification for each specific application [50]. As for cobalt, the information is even more contradictory [36]. Even in the absence of stress exposure (in the control), treatment with cobalt and boron nanoparticles led to an increase in plant growth processes in seedlings of both soft and hard wheat. It is likely that due to the high sowing qualities of the grains used in the experiment, treatment with nanoparticles only slightly increased germination. When using grains of a lower class and with worse germination rates, one can expect higher rates than in the control. During flooding, treatment with both boron and cobalt nanoparticles reduced the negative impact of the stressor. According to other morphometric parameters of seedlings, a stable positive effect of nanoparticles was noted both in variants with and without flooding. Both types of nanoparticles studied were effective. In most cases, no significant differences were found in the effectiveness of the two types of nanoparticles. The photosynthesis rates can be indirectly assessed by the chlorophyll content, which was significantly higher than in the control in both soft and hard wheat plants. Flooding resulted in a significant decrease in chlorophylls a and b in untreated plants, but maintained this indicator in treated ones, helping to maintain productivity. It can be assumed that the positive effect of nanoparticles is associated with universal mechanisms of stress resistance.

The prolonged effect of pre-treatment with nanoparticles can be positive when growing plants under adverse conditions. However, under optimal growing conditions, the positive effect obtained at early stages of development will allow us to preserve more viable plants, and in case of repeated flooding, it will improve the condition of flag leaves, balancing the loss of yield. The data obtained indicate that treatment with boron nanoparticles is more preferable than cobalt nanoparticles and has potential in areas of unstable agriculture with the possibility of temporary flooding. The positive effects were more pronounced for bread wheat, while for hard wheat, the positive effects at the germination stage may be offset at later stages.

## 4. Materials and Methods

### 4.1. Production and Characterization of Nanoparticles

Nanoparticles were obtained by high-temperature condensation using the Migen-3 (Institute for Energy Problems of Chemical Physics, Moscow, Russia) device as described previously [26]. The structure and morphology of cobalt particles were characterized using X-ray powder diffraction and scanning electron microscopy separately [51]. Additional characteristics of the measurements and composition of the nanoparticles used in this study are provided in the Supplementary Materials (Figure S3).

### 4.2. Plant Material

Grains (kernels) of spring hard wheat (*Triticum durum* Desf.) cv Tselinnitsa and spring soft wheat (*Triticum aestivum* L.) cv Orenburgskaya 23 were selected and for all experiments. Grains were received from the breeding center of the Federal Scientific Center of BST RAS (Orenburg, Russia). The grains (kernels) of two types of wheat used in the study belonged to the elite class and had 100% germination under control conditions. Before the experiment, the kernels were surface sterilized in a 2% chlorine solution for 5 min, and then they were washed several times with distilled water until all traces of the disinfectant solution were removed.

### 4.3. Seed Treatment

Suspensions of cobalt and boron nanoparticles were prepared by dispersing an accurately weighed amount of powders in distilled water using an ultrasonic disintegrator UP50H (Hielscher Ultrasonics, Esquire Biotech, Teltow, Germany) in the mode of 3 times for 30 s (0.5 A, 44 kHz) while cooling the container by placing it in ice. The prepared suspension of powders was introduced into a polymer composition of sodium carboxymethylcellulose and polyethylene glycol 400, and was added to an aqueous solution of sodium ethylenediamine tetraacetic acid at a concentration of 0.00037% and dispersed again. The finished preparation was applied to dry kernels (00 on the BBCH (Biologische Bundesanstalt, Bundessortenat and Chemical Industry) scale) at a rate of 5 mL per 100–150 kernels. As a result of the treatment, a film was formed on the surface of the wheat kernels, the characteristics of which are given in the work [52]. The kernels of the control group with the suspensions of cobalt and boron nanoparticles with a concentration of 10^−8^% were treated.

### 4.4. Methodology of Two-Factor Experiments

To study the effect of seed priming with boron and cobalt nanoparticles at the germination (05–09 on the BBCH scale) and tillering (21–23 on the BBCH scale) stages on the resistance of wheat plants to flooding, a two-factor system was used (Table S1). Factor A represented the treatment of wheat seeds with boron and cobalt nanopreparations. Untreated seeds served as the control (no treatment). Factor B represented the imitation of flooding conditions for soft and hard wheat grains at the germination stage in Petri dishes (Experiment 1) and at the tillering stage, when cultivated in a pot culture in a closed synergotron system (Experiment 2). There was no flooding under control conditions (Scheme S1). The number of replicates is indicated for each experiment.

### 4.5. Determination of Flooding Resistance at the Germination Stage

To simulate the field conditions of flooding of freshly sown grains, a method was used in which the grains were in conditions of excess moisture during germination. The grains (kernels) were soaked (immersed in water, the water layer did not exceed 3–4 cm) for 2 days, and then laid out on filter paper in a Petri dish. In the control variant (without flooding), moistened kernels were used. Germination was carried out in Petri dishes in three replicates with 15 kernels in each dish for three experiments. Filter paper in 4 layers was used as a substrate, moistening it to full moisture capacity with distilled water as it dried. Petri dishes were placed in a thermostat for germination at 20–22 °C in darkness. Fully germinated grains were considered to be those that had formed an epicotyl and the first leaf had a well-developed primary embryonic root and at least two adventitious roots formed at the first stage of germination. The indicators were recorded on the 6th day. The number of initial and repeated results does not lead to deviations from generally accepted norms, but ensures statistical reliability of the results. To determine resistance to flooding (anaerobic stress), the resistance index was calculated as the percentage ratio of grain germination in the experimental and control variants.

### 4.6. Determination of Flood Resistance at the Tillering Stage

Cultivation was carried out in a closed system “Sinergotron ISR 1.01” (Zolotoi Shar, Moscow, Russia) with a controlled microclimate. To provide watering, a previously tested optimized nutrient solution was used (Scheme S2). The lighting regime was 18 h per day in the light and 6 h in the dark with a radiation intensity of about 250 μmol m^−2^ s^−1^. The irradiation spectra were determined using a PG 100 N spectrometer from UPRtek (Taibai, Taiwan, Republic of China) (Figure S4, Table S2).

For the flooding experiment, a synergotron chamber was used for normal humidification conditions, and another one was used to avoid the indirect influence of air humidity fluctuations associated with excess water in the chamber. The plants were grown in 75 × 75 × 125 mm containers filled with a mixture of coconut substrate and agroperlite in a ratio of 1:0.5 *v*/*v*. After the seedlings appeared, one plant was left in each container. Watering was carried out with a hydroponic nutrient solution, maintaining the planned substrate moisture (with standard humidification; this is 65–75% of the full moisture capacity). The experiment was performed in triplicate; the number of plants was 10 accessions of each variant. Flooding was carried out at the tillering stage (21–23 on the BBCH scale). After the earing stage, the wheat plant analysis was carried out (73–77 on the BBCH scale).

### 4.7. Morphometric Characteristics of Wheat Plants

The following biometric parameters were determined: plant height, length of the upper internode of the main stem, proportion of plants with spikes (if there is at least one ear on the plant), average number of spikes per plant (all plants in the experiment were used to calculate the average value, including those without spikes), hyperspectral analysis of leaves and pigment analysis.

### 4.8. Hyperspectral Analysis

The data were obtained using hyperspectral imaging (HSI) with the hyperspectral research Module M.Gk. Synergotron hyperspectral camera (Zolotoi Shar, Moscow, Russia). An analysis of images of flag leaves of plants grown from seeds treated with boron and cobalt nanoparticles and exposed to flooding was used. The same leaves were used for subsequent sampling for spectrophotometric analysis of pigment content. A series of images of wheat flag leaves were obtained. To process the obtained data, the normalized vegetation index (NDVI) was used [53].

The index was the difference between two reflectance values divided by the sum of these same values. The formula for the NDVI index is as follows:

Index = (ρλ1 − ρλ2)/(ρλ1 + ρλ2)

NDVI = (ρ800 − ρ680)/(ρ800 + ρ680)

where λ denotes specific wavelengths, ρ denotes the reflectivity, and the numbers 1 and 2 denote the different wavelengths used for each metric. The normalized difference method helps to eliminate the influence of atmospheric scattering, increasing the sensitivity of indices to vegetation characteristics. The index values range from −1 to 1. In our case, the NDVI values range from 0.5 to 0.95. The NDVI is primarily sensitive to chlorophyll content and is widely used to estimate weight, leaf area index, and total vegetation cover [54,55].

By comparing the obtained images with the heat maps superimposed on them, we can conclude that the NDVI helps to clearly separate the background (non-living objects located behind the biological object under study), but also allows us to identify pronounced areas of active metabolism provided by photosynthetic processes in plant leaves.

### 4.9. Biochemical Analysis

The content of chlorophylls and carotenoids (mg L^−1^ of fresh weight) was measured by the standard method using a Genesis 20 spectrophotometer (ThermoSpectronic, Sandy Spring, MD, USA) following the Lichtenthaler method [56]. Leave samples (200 mg) were homogenized in 96% ethanol, after which the absorption was measured at 663 nm and 644 nm for chlorophylls and 452.5 nm for carotenoids. The following formulas were used for calculations:

Chlorophyll a = 10.3 × Abs663 − 0.918 × Abs644;

Chlorophyll b = 9.7 × Abs644 − 3.87 × Abs663;

Carotenoids = 4.2 × Abs452.5 − (0.0264 × chlorophyll a + 0.4260 × chlorophyll b)

### 4.10. Statistical Analysis

Experimental data were statistically compared using analysis of variance (ANOVA) with Statistica v. 12.0 PL (StatSoft, Tulsa, OK, USA) program. The results of physiological and biochemical studies carried out in triplicate were processed using Duncan’s tests with the corresponding errors (*p* ≤ 0.05).

## 5. Conclusions

We have found that the application of boron and cobalt nanoparticles has a positive effect at the seedling stage for both soft and hard wheat plants, especially when exposed to an unfavorable factor—flooding. The treatment increases the number of germinated seeds and their quality indicators. However, it has been shown that long-term prolonged treatment can lead to a decrease in biomass in both species, and can be effective only in the case of repeated negative impact. Thus, it is necessary to further study how important it is to preserve plants at the first stage of development (germination) and whether this compensates for the decrease in biomass productivity established in this work by a large number of plants, which requires field trials. Nanoparticles can have both positive and negative effects on plant development. The increase in chlorophyll may indicate the high potential of cobalt and boron nanoparticles in terms of productivity and compensation for their decline under flooding. The criterion for assessing the state of the flag leaf by the NDVI serves as an additional method for assessing the state of the photosynthetic system and requires further development of the methodological application to assess the correctness of its use in field conditions. It can be assumed that, at least for pre-treatment with boron nanoparticles, improving the state of flag leaves under adverse factors will have prospects in cultivation conditions in regions with a high probability of flooding.

## Data Availability

The original contributions presented in this study are included in the article/Supplementary Materials. Further inquiries can be directed to the corresponding author(s).

## Supplementary Materials

The following supporting information can be downloaded at https://www.mdpi.com/article/10.3390/plants14071044/s1, Figure S1: Histograms of the NDVI of the central part of separated flag leaves of spring soft wheat of cv Orenburgskaya 23; Figure S2: Histograms of the NDVI of the central part of separated flag leaves of spring hard wheat of cv Tselinnitsa; Figure S3: Characteristics of nanoparticles; Table S1: Seed treatments of soft and hard wheat; Scheme S1: Methodology of a comparative two-factor experiment to study the effect of pre-treatment with cobalt and boron nanoparticles on plant development under 20-day root flooding at the tillering stage; Scheme S2: Nutrient solution; Figure S4: Conditions for plant cultivation; Table S2: Lighting conditions in the experiment are given.
