# Supplementary material for: Can Boron and Cobalt Nanoparticles Be Beneficial Effectors to Prevent Flooding-Induced Damage in Durum and Bread Wheat at Germination and Tillering Stage?
Source: Plants (Basel). 2025 Mar 27;14(7):1044. doi: 10.3390/plants14071044 (PMC11990992; doi:10.3390/plants14071044)
Supplement: Supplementary file 1 [file plants-14-01044-s001.zip › plants-3477857-supplementary.pdf]

Supplementary Materials

Supplementary Figure S1

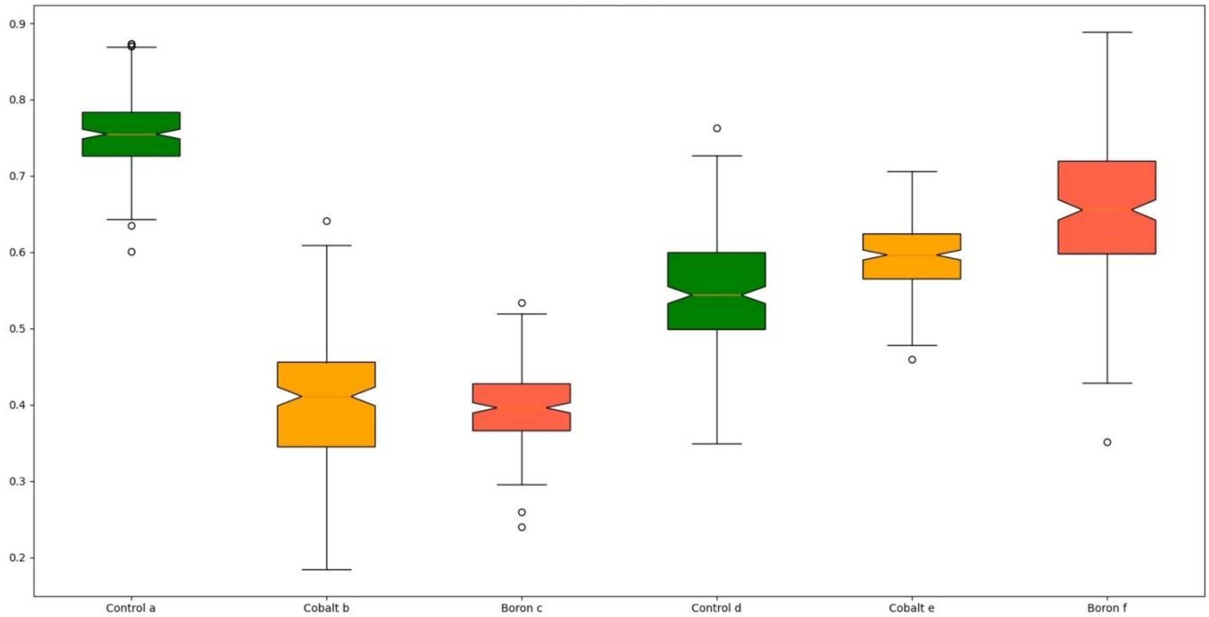

Figure S1. Histograms of the NDVI index of the central part of separated flag leaves of spring soft wheat of the cv Orenburgskaya 23 after pre-treatment of seeds with boron and cobalt nanoparticles under normal conditions and with 20-day root flooding at the tillering stage.

Supplementary Figure S2

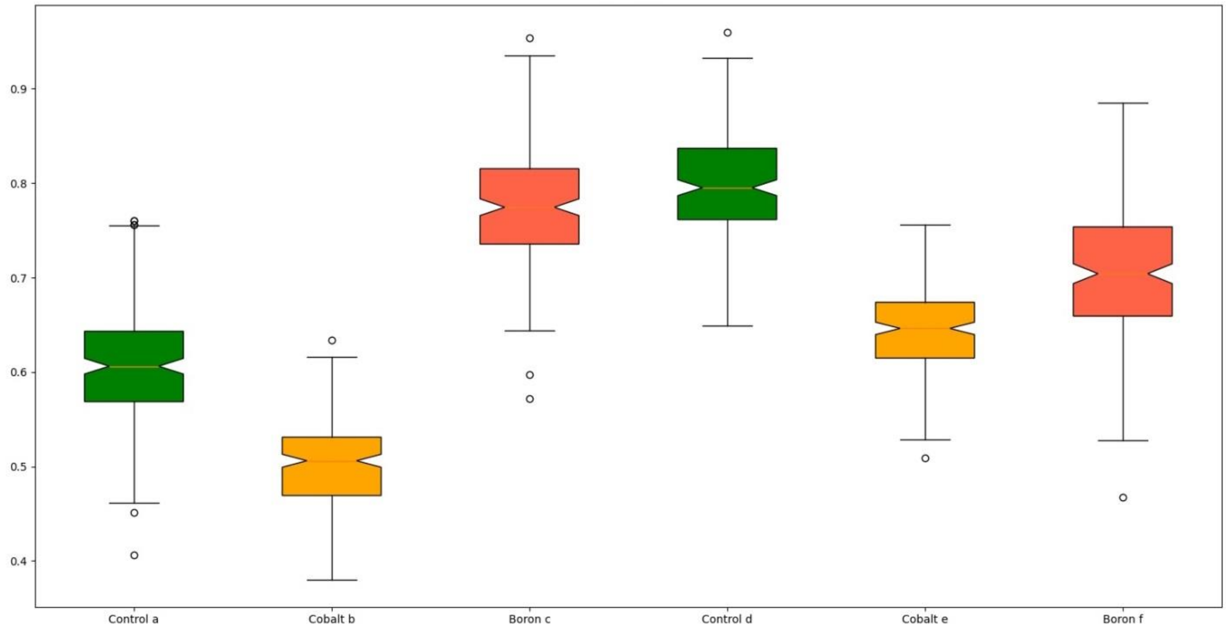

Figure S2. Histograms of the NDVI index of the central part of separated flag leaves of spring hard wheat of the cv Tselinnitsa after pre-treatment of seeds with boron and cobalt nanoparticles under normal conditions and with 20-day root flooding at the tillering stage.

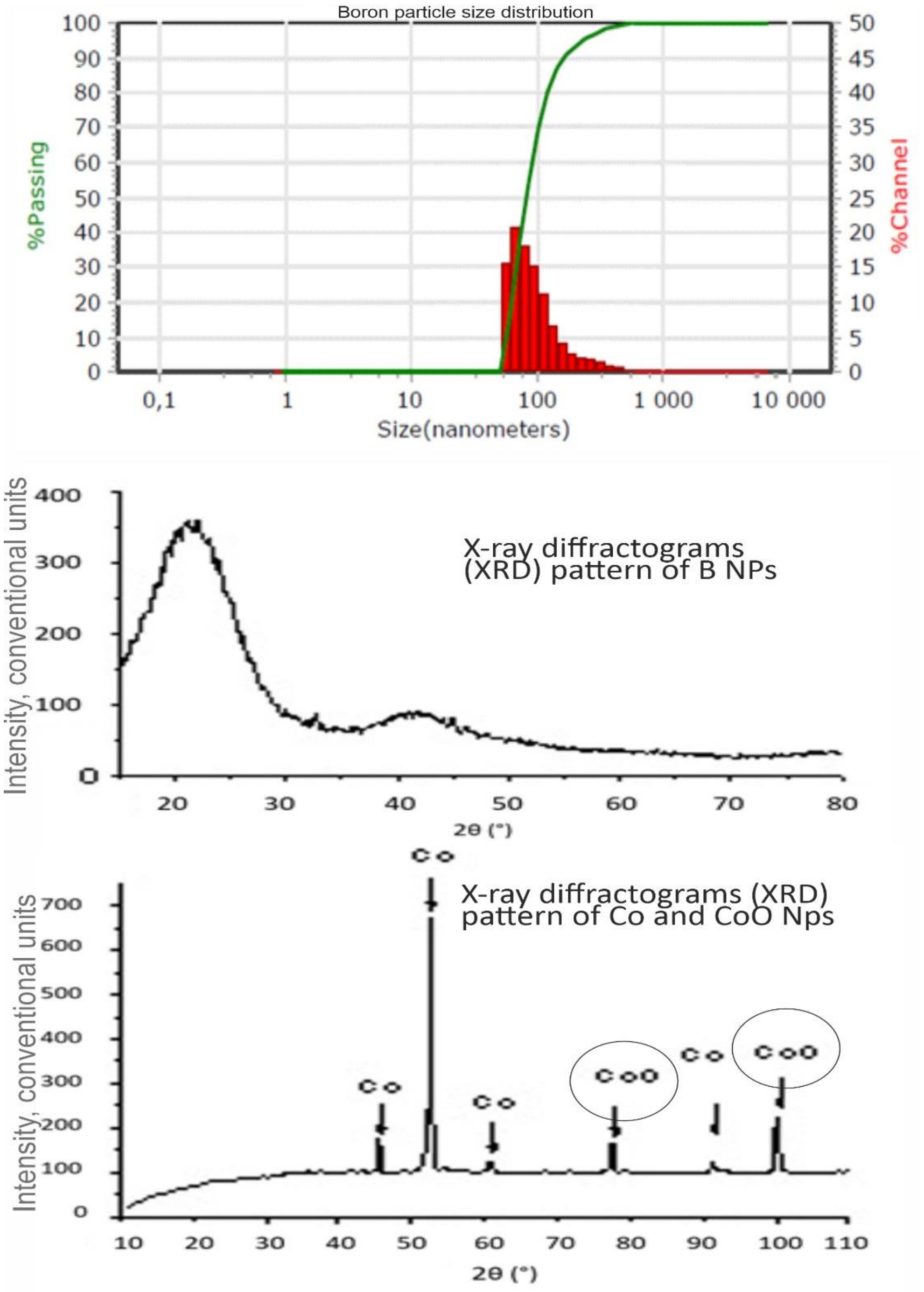

Figure S3. Characteristics of nanoparticles.

Boron nanoparticles were obtained in the center of the Talrose Institute for Energy Problems of Chemical Physics, Russian Academy of Sciences (Moscow, Russia). According to the reports, the maximum size of the primary boron particles used in this work is 134 nm, with 80% of the particles being less than 100 nm and 20% between 100 and 134 nm. Analysis of the boron nanoparticle suspension at a concentration of 10 mg/mL on Microtrack Nanotrack Wave II (Microtrac Inc, USA) showed that the hydrodynamic diameter of the boron nanoparticles was 85.2 nm, as shown in the histogram, the zeta potential is characterized by monodispersity with a single peak at 41.7 mV. Based on these values, we classified it as nanoparticles. The phase purity of boron and cobalt nanoparticles used in this study was confirmed by X-ray diffraction (XRD) analysis and is shown in the corresponding histograms. The absence of additional impurity peaks indicated the phase purity of the nanoparticles.

Table S1. Experimental seed treatments of soft (cv Orenburgskaya 23) and hard (cv Tselinnitsa) wheat used in this study.

№ variant	Cultivar	Moisturizing	Nanoparticle treatment
1	Orenburgskaya 23 (*Triticum aestivum* L.)	Control (without flooding)	Cobalt nanoparticles
2	Orenburgskaya 23 (*Triticum aestivum* L.)		Boron nanoparticles
3	Orenburgskaya 23 (*Triticum aestivum* L.)		Without treatment (control)
4	Tselinnitsa (*Triticum durum* Desf.*)*		Cobalt nanoparticles
5	Tselinnitsa (*Triticum durum* Desf.*)*		Boron nanoparticles
6	Tselinnitsa (*Triticum durum* Desf.*)*		Without treatment (control)
7	Orenburgskaya 23 (*Triticum aestivum* L.)	Flooding	Cobalt nanoparticles
8	Orenburgskaya 23 (*Triticum aestivum* L.)		Boron nanoparticles
9	Orenburgskaya 23 (*Triticum aestivum* L.)		Without treatment (control)
10	Tselinnitsa (*Triticum durum* Desf.*)*		Cobalt nanoparticles
11	Tselinnitsa (*Triticum durum* Desf.*)*		Boron nanoparticles
12	Tselinnitsa(*Triticum durum* Desf.*)*		Without treatment (control)

The experiment was performed in triplicate, the number of plants was 10 accessions of each variant.

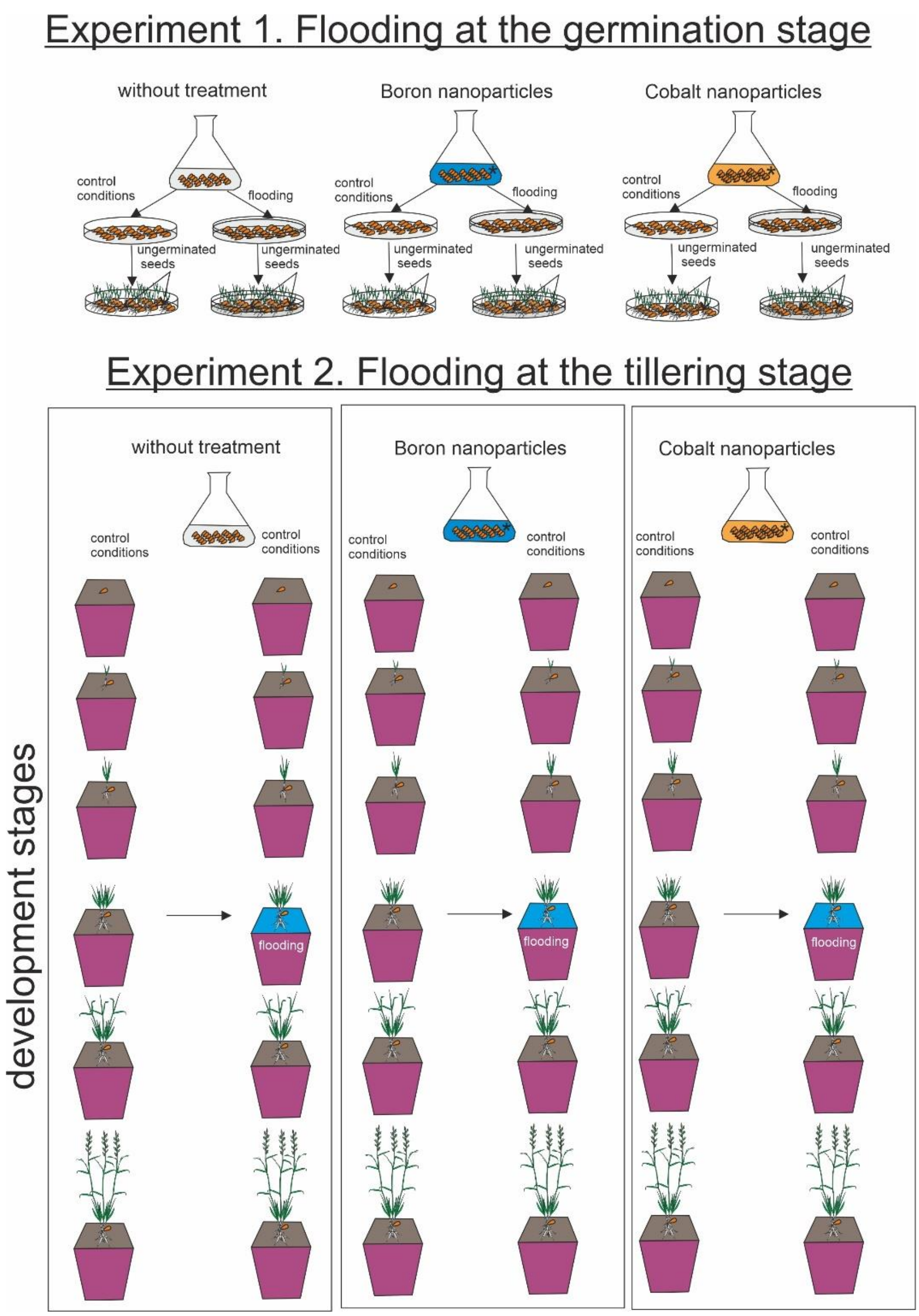

Scheme S1. Methodology of a comparative two-factor experiment to study the effect of pre-treatment with cobalt and boron nanoparticles on wheat plant development under 2-day flooding at the germination stage (Experiment 1) and 20-day root flooding at the tillering stage (Experiment 2).

In the tillering phenophase, some plants were flooded for 20 days according to the experimental design, and standard moisture (65-75% of the maximum permissible moisture content) was maintained the rest of the time. In the control, standard moisture was maintained throughout the entire growing period.

Supplementary Scheme S2.

Nutrient solution: (in mg/l): N-NH4 – 5; N-NO3 -140, P – 41; K – 275; Ca – 100; Mg – 24; S – 30; Fe - 0.94; Mn - 0.14; B - 0.16; Cu - 0.03; Zn - 0.13; Mo - 0.03; pH 5.5-6.0. Fertilizers from the Buysky Plant (Russia, Buy, Kostroma Region) were mainly used as initial salts for the preparation of the solution.

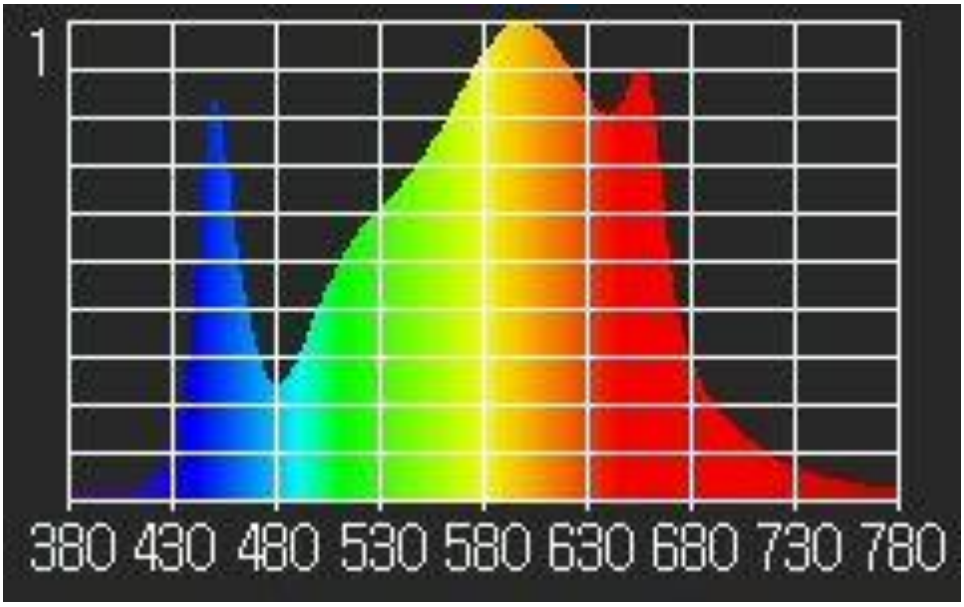

Figure S4. Conditions for plant cultivation.

Conditions for plant cultivation in a closed system synergotron ISR 1.01 (Zolotoy shar, Moscow, Russia). The illumination regime was 18 hours per day in the light and 6 hours in the dark at an irradiation intensity of about 250 μmol m^{-2} s^{-1}. The irradiation spectra were determined on a PG 100 N spectrometer from UPRtek, Taiwan.

Supplementary Table S2. Lighting conditions in the experiment are given in**.**

variant	μmol /m^2*s
ppfd	250,9
Pfd, including:	260,5
UV 380 nm (380-400 nm)	0,27
Blue 440 nm (400-500 nm)	35,25
Green 525 nm (500-600 nm)	103,5
Red 660 nm (600-700 nm)	112,1
Far red 730 nm (700-780 nm)	9,36

Temperature 22-24 C during the day and 17-19 C at night, air humidity 60-65%. Ventilation and air exchange were provided automatically by two fans at the inlet and outlet of the working chamber.
